# The effect of an electronic module about drug abuse prevention on teachers’ beliefs in Indonesia

**DOI:** 10.12688/f1000research.17628.1

**Published:** 2019-01-29

**Authors:** Ghozali Ghozali, Ahmad Azuhairi A, Nor Afiah Mohd Zulkefli, Faisal Ibrahim

**Affiliations:** 1 Department of Community Health, Universiti Putra Malaysia, Serdang, Selangor, 43400, Malaysia

**Keywords:** electronic module, teachers’ beliefs, drug abuse, prevention

## Abstract

**Background:** Drug abuse is a serious global health problem. Globally, 210 million people use illicit drugs each year, of which 200,000 die from drugs. Evidence shows that most drug addicts start using drugs in adolescence (<15-years-old). Adolescents need role models who are able to guide them; teachers have important roles as they are primary role models for students. Therefore, teachers should have positive beliefs to guide students effectively, i.e. they should have good awareness about the threat of drug abuse and high confidence to implement required prevention. This research developed an alternative electronic delivery method of learning material to empower teachers in preventing drug abuse. This study aimed to compare the effect of the electronic and a printed teaching module on teachers’ beliefs about drug abuse prevention.

**Methods:** 260 junior high school teachers were selected randomly. These teachers were split into intervention and control groups. Before intervention, a questionnaire was completed by both groups. The teachers then completed the learning material: electronic module in intervention group and printed module in control group. One month later, data was collected from both groups using the same questionnaire to assess the beliefs of the teachers

**Results:** There was significant positive effect on teachers’ beliefs, both in the intervention and control groups. All categories of beliefs at one month after intervention were significantly higher than those at baseline (P<0.001). Based on between group comparison analysis of mean changes, perceived susceptibility in the intervention group was significantly higher than the control group (P<0.001), while perceived severity, benefits, barriers and efficacy were not significantly different (P>0.05).

**Conclusions:** Electronic and printed module intervention significantly increased teachers’ beliefs in drug abuse prevention. The printed module was still effective to be used as learning media, while the electronic module was an alternative with some advantages.

## Introduction

Drug abuse is a global health problem. Globally, 210 million people in the world use illicit drugs each year, and almost 200,000 of them die as a result of drug use ^1^. The United Nations Office on Drugs and Crime (UNODC) also reported that in 2013, around 246 million people in the world or 1 in 20 people aged 15 to 64 years abused drugs. This was increased from ~3 million individuals from 2012. Approximately 12.19 million people out of 246 million were injecting drug users; 1.65 million individuals with HIV/AIDS ^1^. In a study of HIV testing experience of drug users in Bali, there is evidence that most of the subjects in this study (60%) started using drugs in junior high school, aged 15-years-old or less ^2^.

Adolescence is a critical period because in this period there will be many changes in individual development, physically, psychologically and socially. In this period, adolescents will undergo many conflicts, including between the need for self-control and the need to be independent. In these conditions, adolescents require other persons to guide them. There seems to exist a general consent that education, and teachers in particular, have an important role to play by imparting knowledge, values and skills, as well as by acting as role models for students. Teachers should have adequate preparation to play their role sufficiently and effectively, which involves a combination of cognitive and practical knowledge and skills, values, motivation and attitudes. The lack of this set of preparation may impart a bad influence on the students’ learning outcomes and also students’ behaviors.

Teaching is one enabling and reinforcing factor of student’s behavior, including student’s health behavior ^3^. Positive beliefs of teachers are needed in their role to reinforce factors and be good models for students, including in drug abuse prevention. Hanley *et al.* studied the influence of teacher training on the fidelity of substance use prevention programs implementation in the United States. This study concluded that teacher training on this subject significantly increased the fidelity of implementation of the prevention program ^4^.

Nowadays in Indonesia and many other developing countries, in the field of media and health promotion methods on drugs and the prevention of drug abuse, it is still very rare to find media or methods of delivering messages about the danger of drug abuse and the importance of its prevention that utilizes technological advances, especially for specific individuals like teachers. The electronic messaging media are mostly just short messages via television or radio for universal targets. Media with more complete message content in the form of books or printed brochures generally only target teenagers and general society. Macedo-Rouet *et al*. concluded that there was a strong motivation for using electronic books, and the level of users’ satisfaction was generally very high with this type of medai ^5^. Many studies have also showed that the positive valuation towards electronic books was due to their accessibility and availability ^6, 7^. Shelburne ^8^ stated that the increased availability of electronic books has influenced students’ perception and students value electronic books more than printed ones.

This research developed an alternative electronic delivery method of learning media to empower teachers in preventing drug abuse in the school setting. This study aimed to compare the effect of the electronic module with a printed module on teachers’ beliefs in drug abuse prevention among students.

## Methods

### Study design

This study used comparative design to determine the effects of educational intervention using drug abuse prevention module towards changing and improving teachers’ beliefs in drug abuse prevention. The study was conducted from 10 ^th^ October to 5 ^th^ December 2016. In this study, the intervention group was given an educational intervention using an electronic module, and the control group received the usual printed module with the same content. Before the intervention, a pre-test was conducted to measure teachers’ beliefs. A post-test to measure teachers’ beliefs was conducted at one month after intervention. Pretest and post test used the same instrument.

### Study participants

The final participants of this study were 260 junior high school teachers in Balikpapan, Indonesia. The study sample size was calculated using Jekel’s formula ^9^. The minimum sample size resulted from the calculation was 46 subjects per-group. In accordance with design effect, this number was multiplied by 2; therefore, the minimum number was 92 subjects per-group. With the consideration of 20% attrition rate, the number of expected sample was 115 persons per-group or 230 persons for both groups.

A cluster random sampling was used in this study to select 6 schools from the total of 22 junior high schools. The inclusion criteria of participants were teachers with permanent and full-timer status, and all teachers who signed a written consent form to participate in the study. A total of 278 teachers met the inclusion criteria of this study, from 6 schools selected. Then, the random number assignment was used to allocate each selected school to intervention or control groups.

To prevent selection bias, a researcher assistant performed the allocation process with the instruction that they had to allocate participants to group 1 and group 2, without identifying which group is the intervention or control groups. The result of random assignment were three schools in the intervention group and the other three schools in control group, with the total number teacher who agreed to partake in the study being 133 teachers in the intervention group and 145 teachers in the control group. Therefore, 278 teachers in both groups was set as participants in this study, but 18 teachers could not complete the study or dropped-out from the study with various reasons. Therefore, a total of 260 teachers in both groups completed the study: 128 teachers in the intervention group and 132 teachers in the control group.

In the purpose to prevent bias from the participants, this study was set as a single-blinded study, where all of participants was unaware of whether they are in the intervention or control group. All of investigators made an agreement not to inform to participants about which group they were in, throughout the period of the study.

### Electronic and printed module, and questionnaire

This study involved three experts in training module development and two practitioners in drug and drug abuse prevention to determine the validity of the module and questionnaire used in this study. Before being used in this study, the module and questionnaire were pretested in teachers who did not participate in this study to examine the reliability. The values of Cronbach alpha of the questionnaire was 0.701.

The content of the educational module included definition and types of drugs, factors affecting drug abuse, early detection of drug abuse, usual characteristics of drug abuser, effects of drug abuse, strategies of drug abuse prevention, the role of school and teacher in drug abuse prevention, and how to build a free drugs school.

The questionnaire was used to measure teachers’ beliefs about drug abuse and drug abuse prevention. This questionnaire consisted of four statements about perceived susceptibility, four statements about perceived severity, three statements about perceived benefits, three statements about perceived barriers, and seven statements about perceived self-efficacy.

Questionnaire and modules are available: http://doi.org/10.5281/zenodo.2546532 ^10^

### Data analysis

Descriptive statistics was used to analyze sociodemographic variables and component of beliefs from the questionnaire at baseline. Chi-squared and Mann Whitney U test were used to compare these variables between groups at baseline. Paired-t and Wilcoxon signed-rank test were used to determine the effect of intervention in each group, and independent-t and Mann Whitney U test were used to compare effect of intervention between groups. All statistical analysis was performed using SPSS Version 24, with significance level of 95%.

## Ethical approval

This study obtained approval from Balikpapan District Office of Ministry of Education (420/2180/SKT-VIII/2016) and The University Research Ethics Committee of the Universiti Putra Malaysia (UPM/TNCPI/RMC/1.4.18.1 (JKEUPM)/F1). Written informed consent was obtained from the teachers before they were recruited into the study.

## Results

Table 1 describes the sociodemographic characteristics of study participants in each group. Most of the participants in both groups were female and Javanese. The mean of age was 41.53 ± 9.031 years in the intervention group and 43.19 ± 9.167 years in the control group. The mean of duration of work was 15.79 ± 8.890 years in the intervention group and 17.00 ± 9.388 years in the control group. There were no significant differences between intervention and control groups on the mean of age and duration of work, field of teaching, proportion of gender, and ethnicity.

Table 2 describes each category of beliefs mean score from the questionnaire of participants in the intervention and control groups at baseline. There were no significant differences between intervention and control groups on participants’ categories of beliefs at baseline (P value > 0.05). Table 3 describes within group comparison of participants’ beliefs between baseline and one month after intervention. Overall the results indicated that the teachers’ beliefs at one month after intervention were significantly higher compared with baseline, in both groups. Table 4 describes between group comparison of mean changes in each component of beliefs from baseline to one month after intervention. There were significant differences between the intervention and control groups in mean changes of perceived susceptibility from baseline to one month after intervention (P<0.05). There were no significant differences between intervention and control groups in mean changes of perceived severity, benefits, barriers, efficacy, and total beliefs from baseline to one month after intervention (P value: 0.086, 0.283, 0.261, 0.834 and 0.129, respectively).

**Table 1.** Comparison of sociodemographic characteristics between study groups (n=260).

Characteristics	Intervention n=128 f (%)	Control n=132 f (%)	Test value	P value
Gender			x ^2^=0.694	0.405
Male	47 (36.7)	41 (31.1)		
Female	81 (63.3)	91 (68.9)		
Ethnicity			x ^2^=2.656	0.617
Banjar	31 (24.2)	26 (19.7)		
Bugis	12 (9.4)	15 (11.4)		
Java	57 (44.5)	69 (52.3)		
Kutai	10 (7.8)	7 (5.3)		
Others	18 (14.1)	15 (11.4)		
Field of Teaching			x ^2^=3.904	0.973
Bahasa Indonesia	15 (11.7)	18 (13.6)		
English	13 (10.2)	14 (10.6)		
Counseling	9 (7.0)	11 (8.3)		
Science	19 (14.8)	20 (15.2)		
Social	14 (10.9)	16 (12.1)		
Art	10 (7.8)	10 (7.6)		
Islamic Education	7 (5.5)	9 (6.8)		
Math	14 (10.9)	16 (12.1)		
Christian Education	1 (0.8)	2 (1.5)		
Civic Education	16 (12.5)	9 (6.8)		
ICT	2 (1.6)	1 (0.8)		
Sport Education	8 (6.2)	6 (4.5)		
Age	41.53 ± 9.031	43.19 ± 9.167	Z=-1.629	0.103
Work Duration	15.79 ± 8.890	17.00 ± 9.388	Z=-0.990	0.322

*Significant level at P<0.05

**Table 2.** Comparison of participants’ beliefs on drug abuse prevention between study groups at baseline (n=260).

Categories	Intervention n=128	Control Test value n=132		P value
Susceptibility	11.81±2.982	11.55±3.187	t = 0.697	0.486
Severity	17.88±1.607	17.61±1.759	Z=-1.063	0.288
Benefits	11.88±1.495	11.89±1.785	Z=-0.756	0.449
Barriers	9.73±2.257	9.72±1.788	t = 0.058	0.954
Efficacy	26.56±3.804	26.35±3.477	Z=-0.140	0.889
Total beliefs	77.86±6.812	77.11±6.375	t =-0.921	0.358

*Significant at level p<0.05

**Table 3.** Comparison of each component of beliefs between baseline and one month after intervention (n=260).

Categories	Pretest mean±SD	Post test mean±SD	Mean Change	Test value	P value
Susceptibility					
Intervention	11.81±2.982	14.19±2.694	2.38	t= 16.140	<0.001
Control	11.55±3.187	12.55±3.275	1.00	Z=-6.386	<0.001
Severity					
Intervention	17.88±1.607	18.91±1.111	1.03	Z=-7.320	<0.001
Control	17.61±1.759	19.08±0.941	1.47	Z=-7.665	<0.001
Benefits					
Intervention	11.88±1.495	13.31±1.315	1.43	Z=-7.535	<0.001
Control	11.89±1.785	13.04±1.087	1.15	Z=-7.139	<0.001
Barriers					
Intervention	9.73±2.257	12.02±2.250	2.29	Z=-8.541	<0.001
Control	9.72±1.788	11.57±1.086	1.85	Z=-8.736	<0.001
Efficacy					
Intervention	26.56±3.804	29.66±2.762	3.10	Z=-8.755	<0.001
Control	26.35±3.477	29.26±2.781	2.91	t= 11.996	<0.001
Total beliefs					
Intervention	77.86±6.812	88.09±6.980	10.23	Z=-9.784	<0.001
Control	77.11±6.375	85.49±5.643	8.38	t= 22.889	<0.001

*Significant difference at P<0.05

**Table 4.** Between groups comparison of mean changes of each component of beliefs, from baseline to one month after intervention (n=260).

Category	Mean Change	Test value	P value
Susceptibility			
Intervention	2.38	Z=-6.829	<0.001
Control	1.00		
Severity			
Intervention	1.03	Z=-1.716	0.086
Control	1.47		
Benefits			
Intervention	1.43	Z=-1.073	0.283
Control	1.15		
Barriers			
Intervention	2.29	Z=-1.123	0.261
Control	1.85		
Efficacy			
Intervention	3.10	Z=-0.209	0.834
Control	2.91		
Total beliefs			
Intervention	10.23	Z=-1.517	0.129
Control	8.38		

*Significant difference at P<0.05

## Discussion

The objective of this study was to evaluate the effects of educational intervention for teachers using a printed and electronic module on drug abuse prevention among junior high school students. The intervention group in this study received the educational intervention using an electronic module which has developed by the researchers. The control group received the usual printed module, which contained the same materials as the electronic module.

The findings of this study showed that there was a significant positive effect of the intervention on teachers’s beliefs about drugs and drug abuse prevention, both in the intervention group and the control group. All categories of beliefs at one month after intervention were significantly higher than those at baseline condition (P<0.001), both in intervention and control groups. Based on the between groups comparison analysis of mean changes from baseline to one month after intervention, there was found that mean changes of perceived susceptibility in the intervention group was significantly higher than control group (P<0.001). The mean changes of perceived severity, benefits, barriers, efficacy and total beliefs were not significantly different between intervention and control group (P>0.05). Based on these findings, we conclude that there was almost the same effects of the electronic module and the printed module on all categories of teachers’ beliefs in drug abuse prevention.

Similarly to these findings, Dusenbury *et al*. ^11^ carried out a study to examine the influence of training on teacher beliefs and perceptions about norm setting and student drug use. They found that there was a significant pretest-to-posttest improvement on teacher beliefs and perceptions for several items. There was a significant improvement in teachers expectations that students to not go on to use substances (t=3.391, p=0.001); all teachers better understood how to develop lesson plans for drug education (t=5.886, p<0.001); and finally all teachers had marked improvement in their confidence to use norm setting in teaching (t=9.018, t<0.001). Our findings were also in line with a previous study which found that there was a significant correlation between knowledge on risk factors and the general score of attitude (r Pearson= 0.373, p < 0.001) ^12^. This means that by giving specific knowledge, a person’s attitude toward specific issues will be improved. Belief is a form of attitude.

Azwar ^13^ stated that media is one of the factors influencing the formation of a closed response. Media has a fundamental task in the delivery of information. Media provides information and messages that contain suggestions which will direct one's opinion. When strong enough, the messages brought by the information will provide an effective basis for judging things, so that certain beliefs are formed. In this study, the module which was used in the intervention and control groups is described as learning media. This finding was also similar with that found by Mahmoodabad *et al.* ^14^, who concluded that there was significant improvement on health belief model components average scores among male students two months after receiving an educational intervention about preventive drug dependency in Iran.

## Conclusions

Educational intervention using an electronic module in the intervention group and printed module in the control group significantly increased teachers’ beliefs in drug abuse prevention among students. The findings also indicated that in general both methods equally gave a positive effect on teachers' beliefs in preventing drug abuse. Therefore, both forms of delivery method of learning materials can be used as methods for teacher empowerment efforts in the prevention of drug abuse. The usual printed module was still effective and relevant to be used as learning media in drug abuse prevention, while the electronic module was an alternative that had some advantages in one category, and additionally is easy to carry everywhere, cheaper in production costs, durable, and environmentally friendly. To the best of our knowledge, there are not many studies that have evaluated the effectiveness of this module in the prevention of drug abuse especially on the target of teachers.

## Data availability

### Underlying data

Zenodo: Dataset, Module and Questionnaire of Teachers’ Beliefs About Drug Abuse, http://doi.org/10.5281/zenodo.2546532 ^10^. Demographic data of participants is contained in ‘DEMOGRAPHIC_DATA.xlsx’. Data of comparison of sociodemographic characteristics and participants’ beliefs between study groups, within and between group comparison of beliefs changes is contained ‘RAWDATA_VAR.xlsx’.

### Extended data

Questionnaire used to assess teacher’s beliefs about drug abuse prevention: http://doi.org/10.5281/zenodo.2546532 ^10^.

Electronic/printed module used for the intervention and control groups: http://doi.org/10.5281/zenodo.2546532 ^10^.

Underlying and extended data are available under the terms of the Creative Commons Attribution 4.0 International license (CC-BY 4.0).
