# The effect of electronic and printed module about drug abuse prevention on teachers’ beliefs in Indonesia

**DOI:** 10.12688/f1000research.17628.2

**Published:** 2020-09-17

**Authors:** Ghozali Ghozali, Ahmad Azuhairi A, Nor Afiah Mohd Zulkefli, Faisal Ibrahim

**Affiliations:** 1 Department of Public Health, Universitas Muhammadiyah Kalimantan Timur, Samarinda, 75123, Indonesia; 2 Department of Community Health, Universiti Putra Malaysia, Serdang, Selangor, 43400, Malaysia

**Keywords:** electronic module, teachers’ beliefs, drug abuse, prevention

## Abstract

**Background:** Drug abuse is a serious global health problem. Globally, **269 **million people **or 5.3 percent of the population aged 15‒64 years used **drugs **in 2018**. Evidence shows that most drug addicts start using drugs in adolescence (<15-years-old). Adolescents need role models who are able to guide them; teachers have important roles as they are primary role models for students. Therefore, teachers should have positive beliefs to guide students effectively, i.e. they should have good awareness about the threat of drug abuse and high confidence to implement required prevention. This research developed an alternative electronic delivery method of learning material to empower teachers in preventing drug abuse. This study aimed to compare the effect of the electronic and a printed teaching module on teachers’ beliefs about drug abuse prevention.

**Methods:** 260 junior high school teachers were selected randomly. These teachers were split into **two** groups. Before intervention, a questionnaire was completed by both groups. The teachers then completed the learning material: electronic module in the **first** group and printed module in the **second** group. One month later, data was collected from both groups using the same questionnaire to assess the beliefs of the teachers

**Results:** There was significant positive effect on teachers’ beliefs, both in **electronic module** and **printed module** groups. All categories of beliefs at one month after intervention were significantly higher than those at baseline (P<0.001). Based on between group comparison analysis of mean changes, perceived susceptibility in **electronic module** group was significantly higher than **printed module** group (P<0.001), while perceived severity, benefits, barriers and efficacy were not significantly different (P>0.05).

**Conclusions:** Electronic and printed module intervention significantly increased teachers’ beliefs in drug abuse prevention. The printed module was still effective to be used as learning media, while the electronic module was an alternative with some advantages.

## Introduction

Drug abuse is a global health problem. Globally, the total numbers and the prevalence of drug users in the world have been on the rise, especially in developing countries. In 2009, it was estimated that there were 210 million users or the equivalent of 4.8 percent of the world's population aged 15‒64 years, while in 2018 it was estimated that there were 269 million users or 5.3 percent of the population ^1^. The increase in drug users was much faster in developing countries than in developed countries. This was partly due to the difference in the growth of the population of adolescents and young adults, which made up the largest proportion of those who using drugs, which grew by about 16 percent in developing countries and decreased by 10 percent in developed countries in the 2000–2008 period ^1^. In a study of HIV testing experience of drug users in Bali, there is evidence that most of the subjects in this study (60%) started using drugs in junior high school, aged 15-years-old or less ^2^.

Adolescence is a critical period because in this period there will be many changes in individual development, physically, psychologically and socially. In this period, adolescents will undergo many conflicts, including between the need for self-control and the need to be independent. In these conditions, adolescents require other persons to guide them. There seems to exist a general consent that education, and teachers in particular, have an important role to play by imparting knowledge, values and skills, as well as by acting as role models for students. Teachers should have adequate preparation to play their role sufficiently and effectively, which involves a combination of cognitive and practical knowledge and skills, values, motivation and attitudes. The lack of this set of preparation may impart a bad influence on the students’ learning outcomes and also students’ behaviors.

Teaching is one enabling and reinforcing factor of student’s behavior, including student’s health behavior ^3^. Positive beliefs of teachers are needed in their role to reinforce factors and be good models for students, including in drug abuse prevention. Hanley *et al.* studied the influence of teacher training on the fidelity of substance use prevention programs implementation in the United States. This study concluded that teacher training on this subject significantly increased the fidelity of implementation of the prevention program ^4^.

Nowadays in Indonesia and many other developing countries, in the field of media and health promotion methods on drugs and the prevention of drug abuse, it is still very rare to find media or methods of delivering messages about the danger of drug abuse and the importance of its prevention that utilizes technological advances, especially for specific individuals like teachers. The electronic messaging media are mostly just short messages via television or radio for universal targets. Media with more complete message content in the form of books or printed brochures generally only target teenagers and general society. Macedo-Rouet *et al*. concluded that there was a strong motivation for using electronic books, and the level of users’ satisfaction was generally very high with this type of medai ^5^. Many studies have also showed that the positive valuation towards electronic books was due to their accessibility and availability ^6, 7^. Shelburne ^8^ stated that the increased availability of electronic books has influenced students’ perception and students value electronic books more than printed ones.

This research developed an alternative electronic delivery method of learning media to empower teachers in preventing drug abuse in the school setting. This study aimed to compare the effect of the electronic module with a printed module on teachers’ beliefs in drug abuse prevention among students.

## Methods

### Study design

This study used comparative design to determine the effects of educational intervention using drug abuse prevention module towards changing and improving teachers’ beliefs in drug abuse prevention. The study was conducted from 10 ^th^ October to 5 ^th^ December 2016. The intervention in this study was educational intervention using a specific drug abuse module in electronic form for group 1 and printed form for group 2. Both forms of this module have the same content. Before being given the intervention in the form of modules, a pretest measurement was carried out using a beliefs questionnaire to each of teacher in group 1 and group 2. This pretest was referred to the baseline condition. After the pretest was completed, the module was given to each teacher in the two groups, electronic module for group 1 and printed module for group 2. Each teacher was asked to read and learn the module that has been given completely. In the purpose to avoid interaction bias, each respondent was asked not to provide and distribute the module to others, at least during the study period. One month after the module was given to each teacher, post-test measurement using the same questionnaire was carried out for each teacher in both groups.

### Study participants

The final participants of this study were 260 junior high school teachers in Balikpapan, Indonesia. The study sample size was calculated using Jekel’s formula ^9^. The minimum sample size resulted from the calculation was 46 subjects per-group. In accordance with design effect, this number was multiplied by 2; therefore, the minimum number was 92 subjects per-group. With the consideration of 20% attrition rate, the number of expected sample was 115 persons per-group or 230 persons for both groups.

A cluster random sampling was used in this study to select 6 schools from the total of 22 junior high schools. The inclusion criteria of participants were teachers with permanent and full-timer status, and all teachers who signed a written consent form to participate in the study. A total of 278 teachers met the inclusion criteria of this study, from 6 schools selected. Then, the random number assignment was used to allocate each selected school to group 1 or group 2.

To prevent selection bias, a researcher assistant performed the allocation process with the instruction that they had to allocate participants to group 1 and group 2, without identifying which group is the intervention or control groups. The result of random assignment were three schools in the group 1 and the other three schools in group 2, with the total number of teachers who agreed to partake in the study being 133 teachers in the group 1 and 145 teachers in the group 2. Therefore, 278 teachers in both groups was set as participants in this study, but 18 teachers could not complete the study or dropped-out from the study with various reasons. Therefore, a total of 260 teachers in both groups completed the study: 128 teachers in the group 1 and 132 teachers in the group 2.

In the purpose to prevent bias from the participants, this study was set as a single-blinded study, where all of participants was unaware of whether they are in the intervention or control group. All of investigators made an agreement not to inform to participants about which group they were in, throughout the period of the study.

### Electronic and printed module, and questionnaire

This study involved three experts in training module development and two practitioners in drug and drug abuse prevention to determine the validity of the module and questionnaire used in this study. Before being used in this study, the module and questionnaire were pretested in teachers who did not participate in this study to examine the reliability. The values of Cronbach alpha of the questionnaire were 0.700 for perceived susceptibility, 0.705 for perceived severity, 0.632 for perceived benefit, 0.716 for perceived barriers, and 0.690 for perceived self-efficacy.

The content of the educational module included definition and types of drugs, factors affecting drug abuse, early detection of drug abuse, usual characteristics of drug abuser, effects of drug abuse, strategies of drug abuse prevention, the role of school and teacher in drug abuse prevention, and how to build a free drugs school.

The questionnaire was used to measure teachers’ beliefs about drug abuse and drug abuse prevention. This questionnaire consisted of four statements about perceived susceptibility, four statements about perceived severity, three statements about perceived benefits, three statements about perceived barriers, and seven statements about perceived self-efficacy.

Questionnaire and modules are available: http://doi.org/10.5281/zenodo.2546532 ^10^

### Data analysis

Descriptive statistics was used to analyze sociodemographic variables and component of beliefs from the questionnaire at baseline. Chi-squared and Mann Whitney U test were used to compare these variables between groups at baseline. A one-way ANOVA with repeated measures test was used to determine the effect of intervention in each group, and a Kruskal-Wallis test was used to compare effect of intervention between groups. All statistical analysis was performed using SPSS Version 24, with significance level of P < 0.05.

## Ethical approval

This study obtained approval from Balikpapan District Office of Ministry of Education (420/2180/SKT-VIII/2016) and The University Research Ethics Committee of the Universiti Putra Malaysia (UPM/TNCPI/RMC/1.4.18.1 (JKEUPM)/F1). Written informed consent was obtained from the teachers before they were recruited into the study.

## Results

Table 1 describes the sociodemographic characteristics of study participants in each group. Most of the participants in both groups were female and Javanese. The mean of age was 41.53 ± 9.031 years in the group 1 and 43.19 ± 9.167 years in the group 2. The mean of duration of work was 15.79 ± 8.890 years in the group 1 and 17.00 ± 9.388 years in the group 2. There were no significant differences between groups on the mean of age and duration of work, field of teaching, proportion of gender, and ethnicity.

Table 2 describes each category of beliefs mean score from the questionnaire of participants in the two groups at baseline. There were no significant differences between groups on participants’ categories of beliefs at baseline (P value > 0.05). Table 3 describes within and between group comparison of participants’ mean changes in each component of beliefs from baseline to one month after intervention. Overall the results indicated that the teachers’ beliefs at one month after intervention were significantly higher compared with baseline, in both groups. There was significant difference between groups in mean change of perceived susceptibility from baseline to one month after intervention (P<0.001). The positive mean change and partial eta squared of perceived susceptibility in group 1 were significantly higher than group 2, which were 2.38 and 0.672 compared with 1.00 and 0.274. There were no significant differences between groups in mean changes of perceived severity, benefits, barriers, efficacy, and total beliefs from baseline to one month after intervention (P value: 0.086, 0.283, 0.261, 0.834 and 0.129, respectively).

**Table 1.** Comparison of sociodemographic characteristics between study groups (n=260).

Characteristics	Group 1 n=128 f (%)	Group 2 n=132 f (%)	Test value	P value
Gender			x ^2^=0.694	0.405
Male	47 (36.7)	41 (31.1)		
Female	81 (63.3)	91 (68.9)		
Ethnicity			x ^2^=2.656	0.617
Banjar	31 (24.2)	26 (19.7)		
Bugis	12 (9.4)	15 (11.4)		
Java	57 (44.5)	69 (52.3)		
Kutai	10 (7.8)	7 (5.3)		
Others	18 (14.1)	15 (11.4)		
Field of Teaching			x ^2^=3.904	0.973
Bahasa Indonesia	15 (11.7)	18 (13.6)		
English	13 (10.2)	14 (10.6)		
Counseling	9 (7.0)	11 (8.3)		
Science	19 (14.8)	20 (15.2)		
Social	14 (10.9)	16 (12.1)		
Art	10 (7.8)	10 (7.6)		
Islamic Education	7 (5.5)	9 (6.8)		
Math	14 (10.9)	16 (12.1)		
Christian Education	1 (0.8)	2 (1.5)		
Civic Education	16 (12.5)	9 (6.8)		
ICT	2 (1.6)	1 (0.8)		
Sport Education	8 (6.2)	6 (4.5)		
Age	41.53 ± 9.031	43.19 ± 9.167	Z=-1.629	0.103
Work Duration	15.79 ± 8.890	17.00 ± 9.388	Z=-0.990	0.322

*Significant level at P<0.05

**Table 2.** Comparison of participants’ beliefs on drug abuse prevention between study groups at baseline (n=260).

Categories	Group 1 n=128	Group 2. n=132	Test value	P value
Susceptibility	11.81±2.982	11.55±3.187	t = 0.697	0.486
Severity	17.88±1.607	17.61±1.759	Z=-1.063	0.288
Benefits	11.88±1.495	11.89±1.785	Z=-0.756	0.449
Barriers	9.73±2.257	9.72±1.788	t = 0.058	0.954
Efficacy	26.56±3.804	26.35±3.477	Z=-0.140	0.889
Total beliefs	77.86±6.812	77.11±6.375	t =-0.921	0.358

*Significant at level p<0.05

**Table 3.** Comparison of each component of beliefs between baseline (pretest) and one month after intervention (post test) (n=260).

Categories	Pretest	Post test	Mean	Within Group			Between Groups	
	mean±SD	mean±SD	Change	Test Value	P Value	Partial Eta Squared	Test Value	P Value
Susceptibility								
Group 1	11.81±2.982	14.19±2.694	2.38	F= 260.494	<0.001	0.672	X ^2^=46.637	<0.001
Group 2	11.55±3.187	12.55±3.275	1.00	F=49.406	<0.001	0.274		
Severity								
Group 1	17.88±1.607	18.91±1.111	1.03	F=102.981	<0.001	0.448	X ^2^=2.946	0.086
Group 2	17.61±1.759	19.08±0.941	1.47	F=91.388	<0.001	0.411		
Benefits								
Group 1	11.88±1.495	13.31±1.315	1.43	F=98.944	<0.001	0.438	X ^2^=1.151	0.283
Group 2	11.89±1.785	13.04±1.087	1.15	F=74695	<0.001	0.363		
Barriers								
Group 1	9.73±2.257	12.02±2.250	2.29	F=143.808	<0.001	0.531	X ^2^=1.261	0.261
Group 2	9.72±1.788	11.57±1.086	1.85	F=162.782	<0.001	0.554		
Efficacy								
Group 1	26.56±3.804	29.66±2.762	3.10	F=129.272	<0.001	0.504	X ^2^=0.044	0.834
Group 2	26.35±3.477	29.26±2.781	2.91	F=143.906	<0.001	0.523		
Total beliefs								
Group 1	77.86±6.812	88.09±6.980.	10.23	F=338.442	<0.001	0.727	X ^2^=2.300	0.129
Group 2	77.11±6.375	85.49±5.643	8.38	F=523917	<0.001	0.800		

*Significant difference at P<0.05

## Discussion

The objective of this study was to evaluate the effects of educational intervention for teachers using a printed and electronic module on drug abuse prevention among junior high school students. The group 1 in this study received the educational intervention using an electronic module which has developed by the researchers. The group 2 received the usual printed module, which contained the same materials as the electronic module.

Based on findings of this study, all components of beliefs in both groups at one month after intervention were significantly increased compared to baseline condition before intervention. The only significant difference between electronic and printed module groups was in terms of increased perceived susceptibility, where in the electronic module group there was a significantly higher increase compared to the printed module group.

Similarly to these findings, Dusenbury *et al*. ^11^ carried out a study to examine the influence of training on teacher beliefs and perceptions about norm setting and student drug use. They found that there was a significant pretest-to-posttest improvement on teacher beliefs and perceptions for several items. There was a significant improvement in teachers expectations that students to not go on to use substances (t=3.391, p=0.001); all teachers better understood how to develop lesson plans for drug education (t=5.886, p<0.001); and finally all teachers had marked improvement in their confidence to use norm setting in teaching (t=9.018, t<0.001). Our findings were also in line with a previous study which found that there was a significant correlation between knowledge on risk factors and the general score of attitude (r Pearson= 0.373, p < 0.001) ^12^. This means that by giving specific knowledge, a person’s attitude toward specific issues will be improved. Belief is a form of attitude.

Azwar ^13^ stated that media is one of the factors influencing the formation of a closed response. Media has a fundamental task in the delivery of information. Media provides information and messages that contain suggestions which will direct one's opinion. When strong enough, the messages brought by the information will provide an effective basis for judging things, so that certain beliefs are formed. In this study, the module which was used in both groups is described as learning media. This finding was also similar with that found by Mahmoodabad *et al.* ^14^, who concluded that there was significant improvement on health belief model components average scores among male students two months after receiving an educational intervention about preventive drug dependency in Iran.

According to the importance of alternative, more advanced methods in delivering drug prevention messages, this study was also relevant with Hansen *et al*. who studied about the impact of technological enhancements toward teachers’ attitude in delivering drug abuse prevention program in U.S. This study revealed that teachers who used the technological enhancements found it easier to implement the program compare to others who delivered the program as usual. Furthermore, teachers’ attitude about the program improved after experienced the enhancements and the majority of teachers wishing to continue using them in the future ^15^.

## Conclusions

Educational intervention using electronic and printed module significantly increased teachers’ beliefs in drug abuse prevention among students. The findings also indicated that in general both methods equally gave a positive effect on teachers' beliefs in preventing drug abuse. Therefore, both forms of delivery method of learning materials can be used as methods for teacher empowerment efforts in the prevention of drug abuse. The usual printed module was still effective and relevant to be used as learning media in drug abuse prevention, while the electronic module was an alternative that had some advantages in one category, and additionally is easy to carry everywhere, cheaper in production costs, durable, and environmentally friendly. To the best of our knowledge, there are not many studies that have evaluated the effectiveness of this module in the prevention of drug abuse especially on the target of teachers.

## Data availability

### Underlying data

Zenodo: Dataset, Module and Questionnaire of Teachers’ Beliefs About Drug Abuse, http://doi.org/10.5281/zenodo.2546532 ^10^. Demographic data of participants is contained in ‘DEMOGRAPHIC_DATA.xlsx’. Data of comparison of sociodemographic characteristics and participants’ beliefs between study groups, within and between group comparison of beliefs changes is contained ‘RAWDATA_VAR.xlsx’.

### Extended data

Questionnaire used to assess teacher’s beliefs about drug abuse prevention: http://doi.org/10.5281/zenodo.2546532 ^10^.

Electronic/printed module used for the intervention: http://doi.org/10.5281/zenodo.2546532 ^10^.

Underlying and extended data are available under the terms of the Creative Commons Attribution 4.0 International license (CC-BY 4.0).
