# Supplementary material for: The effect of electronic and printed module about drug abuse prevention on teachers’ beliefs in Indonesia
Source: F1000Res. 2020 Sep 17;8:115. Originally published 2019 Jan 29. [Version 2] doi: 10.12688/f1000research.17628.2 (PMC10521037; doi:10.12688/f1000research.17628.2)
Supplement: Peer review history

# Reviewer report: Reviewer response for version 2

**DOI:** 10.5256/f1000research.29535.r171520

**Published:** 2023-06-27

**Authors:** Adama Grace Ngozi

**Affiliations:** 1 University of Nigeria, Nsukka, Enugu, Nigeria

The keywords should include the modules used in the experiment. Editorial work e.g. “who are using the drugs” instead of “those who using the drugs”.

 Conceptualization of keywords are lacking such as drug abuse, adolescence, adolescent.

 Under method, this is educational intervention using electronic and printed module to increase teachers’ beliefs in drug abuse prevention among students. This is a comparative study and there should be no need to be using a control group. It is just comparing two groups, one group using electronic and another using printed materials to know the effect of efficacy of one over the other.

 Using pre-test and post-test is in order to help ascertain the effect of material used on the teachers.

 Under sampling, only Balikpapan; the reason for selecting this state or town was not stated in the work because I know there may be other states or towns in Indonesia.

 The APA referencing style should be uniform.

Is the work clearly and accurately presented and does it cite the current literature?

Partly

If applicable, is the statistical analysis and its interpretation appropriate?

Yes

Are all the source data underlying the results available to ensure full reproducibility?

Yes

Is the study design appropriate and is the work technically sound?

Partly

Are the conclusions drawn adequately supported by the results?

Yes

Are sufficient details of methods and analysis provided to allow replication by others?

Yes

Reviewer Expertise:

Early intervention in special education

I confirm that I have read this submission and believe that I have an appropriate level of expertise to confirm that it is of an acceptable scientific standard.

# Reviewer report: Reviewer response for version 2

**DOI:** 10.5256/f1000research.29535.r71575

**Published:** 2020-10-15

**Authors:** William B. Hansen

**Affiliations:** 1 Prevention Strategies, LLC, Browns Summit, NC, USA

I have no further comments on the article. The authors were responsive to my critique.

Is the work clearly and accurately presented and does it cite the current literature?

Partly

If applicable, is the statistical analysis and its interpretation appropriate?

Partly

Are all the source data underlying the results available to ensure full reproducibility?

Yes

Is the study design appropriate and is the work technically sound?

Yes

Are the conclusions drawn adequately supported by the results?

Partly

Are sufficient details of methods and analysis provided to allow replication by others?

Yes

Reviewer Expertise:

NA

I confirm that I have read this submission and believe that I have an appropriate level of expertise to confirm that it is of an acceptable scientific standard.

# Response

**Published:** 2020-10-15

**Authors:** Ghozali Ghozali

**Affiliations:** Universitas Muhammadiyah Kalimantan Timur, Indonesia

Thank you for your approval, Dr William B. Hansen

# Reviewer report: Reviewer response for version 1

**DOI:** 10.5256/f1000research.19277.r66158

**Published:** 2020-07-14

**Authors:** Manop Kanato

**Affiliations:** 1 Department of Community Medicine, Khon Kaen University, Khon Kaen, Thailand

Although this article is interesting, minor revision is needed. UNODC world drug report is now 2020, reference WDR 2011 may be too old.

 The article needs more details on the intervention and implementation process. Characteristics of the teacher should be taken into account in the analysis. Statistics regarding covariate need to be considered rather than paired and independent t-test.

Is the work clearly and accurately presented and does it cite the current literature?

Yes

If applicable, is the statistical analysis and its interpretation appropriate?

Partly

Are all the source data underlying the results available to ensure full reproducibility?

No source data required

Is the study design appropriate and is the work technically sound?

Yes

Are the conclusions drawn adequately supported by the results?

Partly

Are sufficient details of methods and analysis provided to allow replication by others?

Partly

Reviewer Expertise:

Drug abuse, Health behavior, Epidemiology, Policy evaluation, Preventive Medicine & Public Health

I confirm that I have read this submission and believe that I have an appropriate level of expertise to confirm that it is of an acceptable scientific standard, however I have significant reservations, as outlined above.

# Response

**Published:** 2020-09-10

**Authors:** Ghozali Ghozali

**Affiliations:** Universitas Muhammadiyah Kalimantan Timur, Indonesia

Dear Dr Manop Kanato,

Thank you very much for reviewing our article and giving some great suggestions to make our article better. We have made corrections according to your suggestions and the revised article has also been uploaded. 

- The first paragraph of the introduction was partly changed according to the newer reference of World Drug Report as suggested. The reference of World Drug Report has been changed to the 2020 version.
- The more detailed explanation in the study design especially to clarify the intervention and implementation process has been added according to the suggestion.
- Characteristics of the teachers analysis were not fully presented in this article because there were too many descriptions, so that the material was being prepared to be made in another separate publication article. This article is focused on the comparison of the effects between the electronic module and the printed module, table 1 on the comparison of the characteristics of teachers between group 1 and group 2 is intended to provide an illustration that in terms of the characteristics of teachers the two groups were comparable.
- The statistical data analysis has been improved according to suggestion. T test, Wilcoxon and Mann Whitney U tests have been replaced with repeated measure ANOVA and Kruskal Wallis Test. Tables 3 and 4 have been combined into table 3 which contains the results of statistical test comparisons within and between groups using repeated measure ANOVA and Kruskal Wallis Tests.

We sincerely hope that the improvements we have made can fulfill the suggestions you have given to make our article better.

Best regards

# Reviewer report: Reviewer response for version 1

**DOI:** 10.5256/f1000research.19277.r45218

**Published:** 2019-03-15

**Authors:** William B. Hansen

**Affiliations:** 1 Prevention Strategies, LLC, Browns Summit, NC, USA

- Correct this grammar:
  with the total number teacher who agreed to partake in the study being 133 teachers in the intervention group and 145 teachers in the control group.
  Should be:
  with the total number of teachers who agreed to partake in the study being 133 teachers in the intervention group and 145 teachers in the control group.
- With a nested design, teachers within schools and schools being assigned to condition, the required number of teachers needed would be greater than the number apparently calculated. I suggest software such as Optimal Design. https://www.theanalysisfactor.com/sample-size-randomized-trials/
- Only one alpha coefficient is reported but it is apparent that there are five subsclasses. Coefficients should be calculated for each separately, unless there is statistical evidence that they all load on one factor. No such evidence is provided. I appreciate that the questionnaire was made available. 
- It is not clear if a pre-test and post-test were given, or if just a post-test was given. It sounds like there might have been a pre-test (baseline is mentioned), but it is not clear. 
- Significance should be p<.05, not 95% (this was correctly noted in the tables, but not in the narrative).
- The data presented in Table 3 is very promising; however, a one-way ANOVA with repeated measures or MANOVA would have been a more appropriate analytic method. Also, it is a bit bothersome to have t values presented some of the time and Z values the rest of the time. If consistency cannot be maintained, it should be explained. 
- Tables 3 and 4 present results that could be combined. 
- The second paragraph of the discussion includes a wordy rehash of the results paragraph about the same topic. I think the discussion should focus more on interpretation of results. 
- There really is not a treatment and a control group. Both sets of teachers are exposed to an intervention; therefore, both groups are treatment groups. They are just different in the format with which they received training. There really is no control group and it is misleading to call the group that received paper instruction as such. I would change the title of the paper to fit this reality.

Is the work clearly and accurately presented and does it cite the current literature?

Partly

If applicable, is the statistical analysis and its interpretation appropriate?

Partly

Are all the source data underlying the results available to ensure full reproducibility?

Yes

Is the study design appropriate and is the work technically sound?

Yes

Are the conclusions drawn adequately supported by the results?

Partly

Are sufficient details of methods and analysis provided to allow replication by others?

Yes

Reviewer Expertise:

drug prevention

I confirm that I have read this submission and believe that I have an appropriate level of expertise to confirm that it is of an acceptable scientific standard, however I have significant reservations, as outlined above.

# Response

**Published:** 2019-04-03

**Authors:** Ghozali Ghozali

**Affiliations:** Universitas Muhammadiyah Kalimantan Timur, Indonesia

Thank you so much for your review and your suggestions. We will make some corrections and explanations based on your suggestions. The corrections will be in our revised article. Some explanations are below:

- The number of teachers recruited in each group has considered the design effect and attrition rate. Based on the minimum sample calculation, the minimum number of samples was 46 per-group, considered the design effect then the number multiplied by 2 to 92 people per-group. Taking into account the attrition rate of 20%, the number of samples was 115 per-group. The actual number of samples recruited at the initial stage was 133 people in group 1 and 145 people in group 2 (greater than the optimum sample size, 115).
- Questionnaire was available at https://zenodo.org/record/2542702#.XKOtEuszYTE
- A pre-test and post-test were given. We mean baseline measurement of variables as a pre-test. Anyway, we will make correction in the narrative.
- We did not use ANOVA or MANOVA since there was only two groups and two times measurement of dependent variable. The t and Z values were retained in table 3 because of the different statistical tests used. The t value was obtained from the paired-t test (parametric) , while the Z value of the Wilcoxon signed rank test (non-parametric). However, we will accommodate suggestions for combining tables 3 and 4 in the revised article.

# Response

**Published:** 2020-09-10

**Authors:** Ghozali Ghozali

**Affiliations:** Universitas Muhammadiyah Kalimantan Timur, Indonesia

Dear Dr William B. Hansen,

 Thank you so much for reviewing our article and giving us some great suggestions. We have carefully considered all your comments and suggestions and we have accommodated them in the revised version of the manuscript we have uploaded. The points of explanation and updates are as follows:

- The grammar of the third paragraph in the section of study participants has been corrected according to the suggestion.
- This study has used a sample size larger than the number apparently calculated. The results of the minimum sample calculation in this study according to the formula and added with the attrition rate were 115 teachers per group or 230 teachers for two groups. The actual number of respondents in this study was 128 for group 1 and 132 for group 2.
- The alpha coefficients have been added for each of the five subclasses of beliefs, as suggested
- In the study design section, there has been made clear that the “pretest” is referred to as “baseline” conditions.
- Significance level in the narrative of data analysis section has been corrected to P < 0.05
- Tables 3 and 4 have been combined into table 3, following the suggestion. Paired t and Wilcoxon tests have been changed with repeated measure ANOVA, so that the statistical values have also been adjusted and consistent. The description of table 3 in the results section has been updated
- The second paragraph in the discussion has been improved, focusing more on the interpretation of the results.
- We agreed with the comment that both groups in our study are treatment groups. The title of manuscript was updated by adding “and printed”. The words of “intervention group” and “control group” have been changed to “group 1” and “group 2” in the whole of the manuscript. 

We sincerely hope that the responses and revisions that we have made can fulfill the suggestions that you have provided to make our article better.

 Best regards.
